# Supplementary material for: Reference Genes Screening and Gene Expression Patterns Analysis Involved in Gelsenicine Biosynthesis under Different Hormone Treatments in Gelsemium elegans
Source: Int J Mol Sci. 2023 Nov 4;24(21):15973. doi: 10.3390/ijms242115973 (PMC10648913; doi:10.3390/ijms242115973)
Supplement: Supplementary file 1 [file ijms-24-15973-s001.zip › ijms-2625526-supplementary.pdf]

Supplementary materials

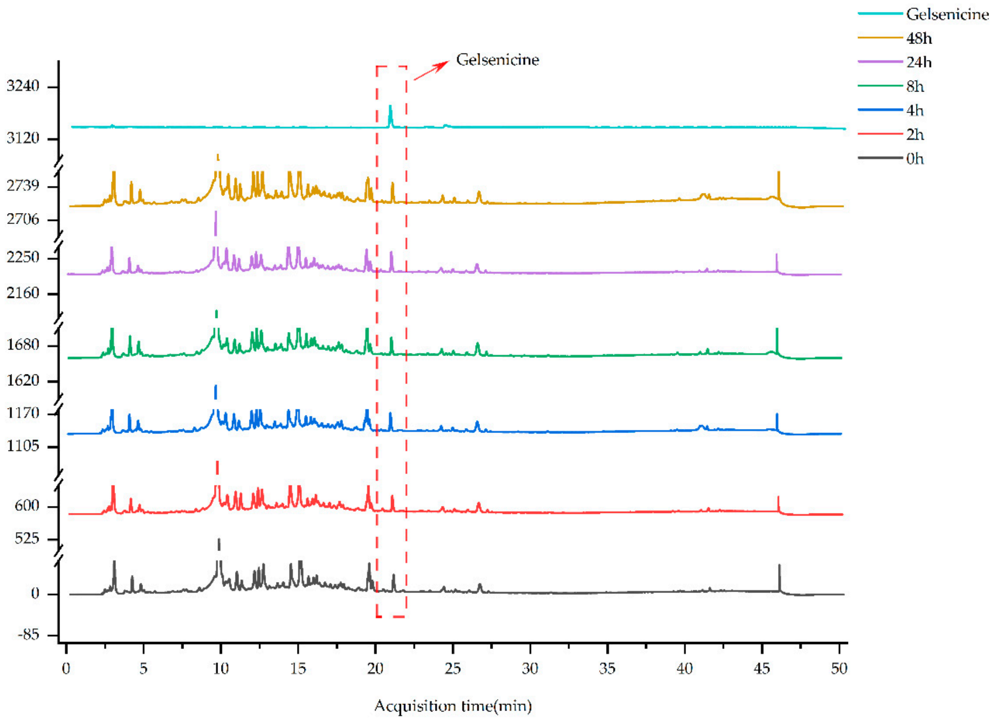

Figure.S1 Chromatogram of gelsenicine under SA treatment for 0-48 hours.

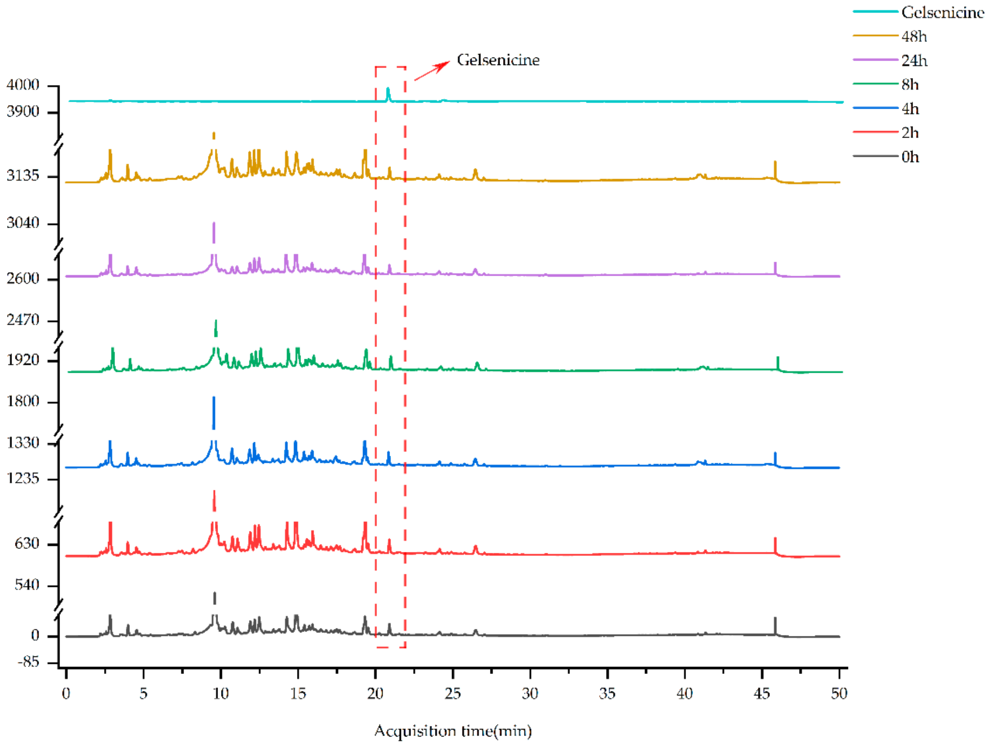

Figure.S2 Chromatogram of gelsenicine under MeJA treatment for 0-48 hours.

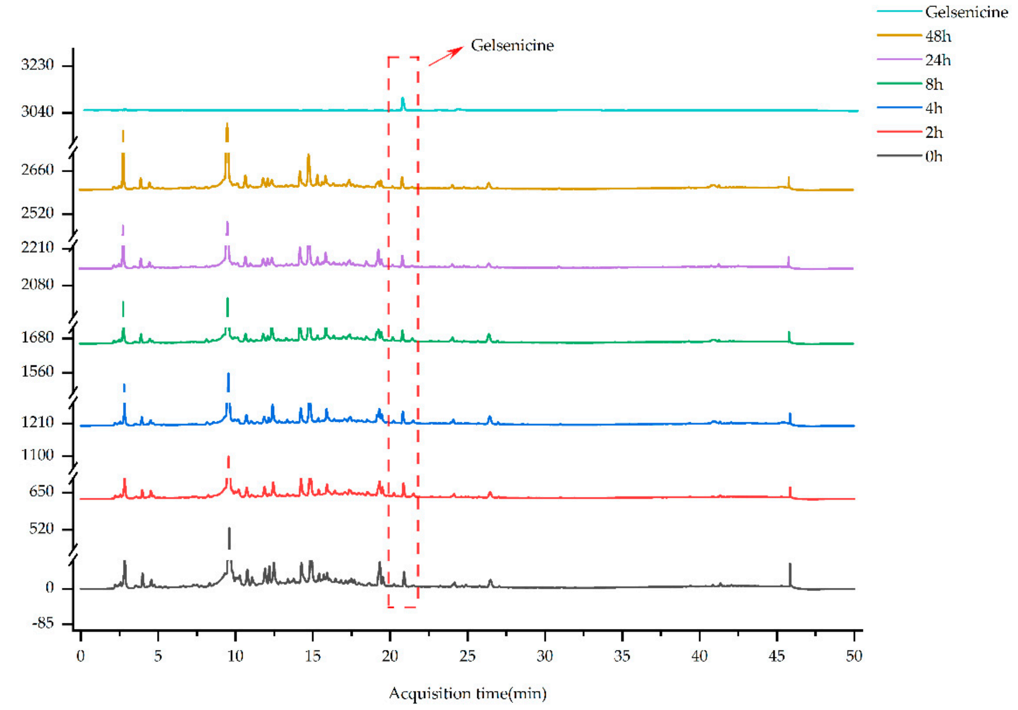

Figure.S3 Chromatogram of gelsenicine under ETH treatment for 0-48 hours.

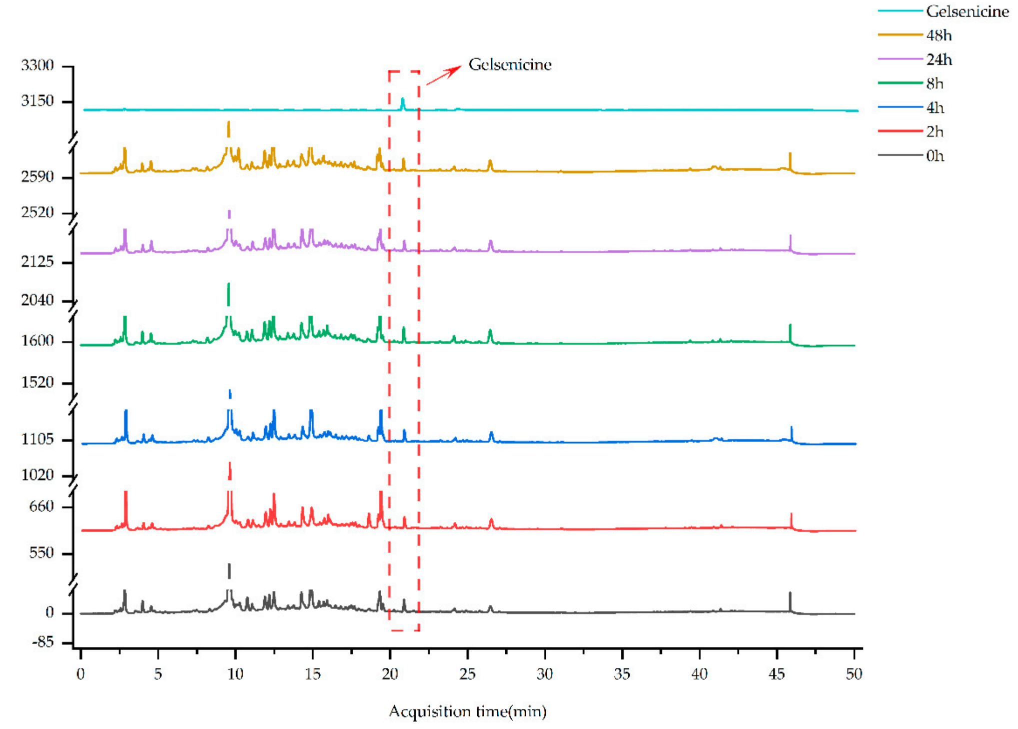

Figure.S4 Chromatogram of gelsenicine under ABA treatment for 0-48 hours.

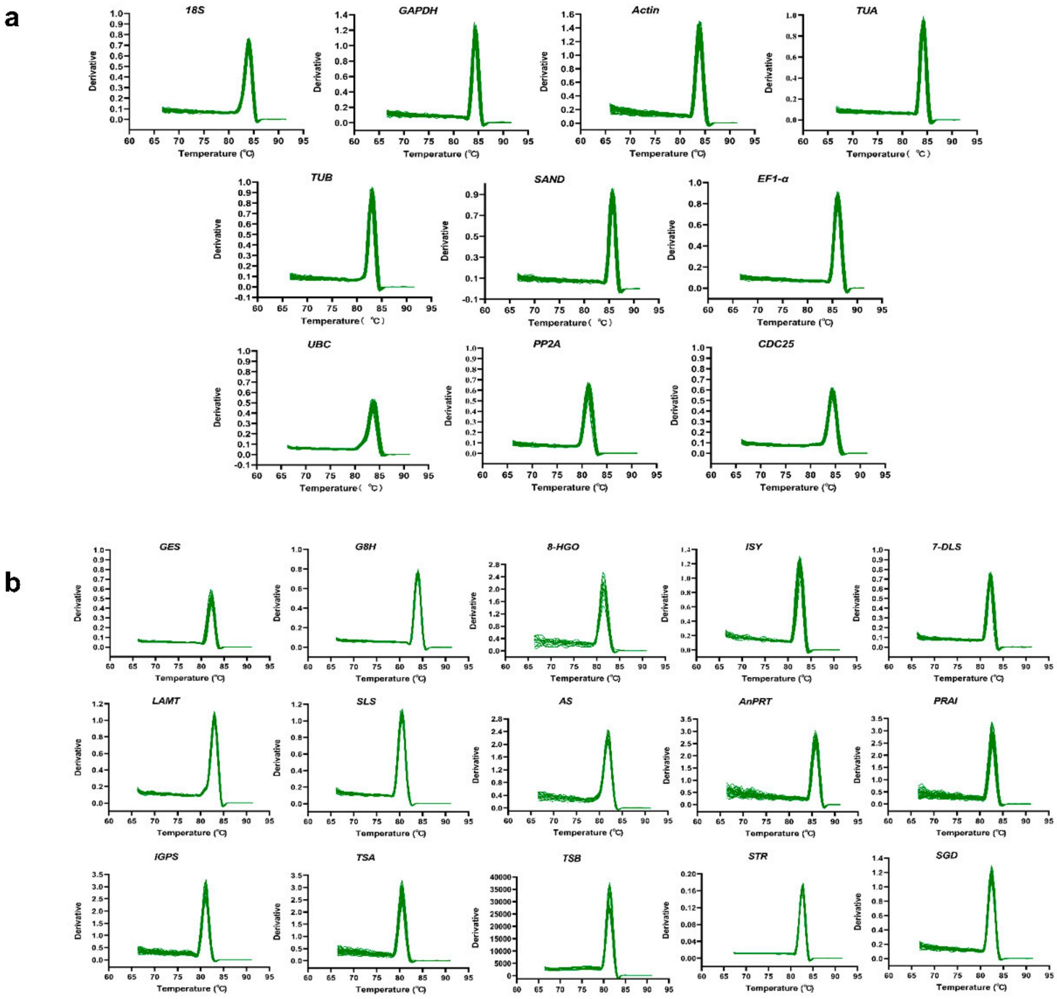

Figure.S5 (a). Melting curve analysis of 10 candidate reference genes. (b). Melting curve analysis of 15 pathway-related genes.
